# SBML2HYB: a Python interface for SBML compatible hybrid modeling

**DOI:** 10.1093/bioinformatics/btad044

**Published:** 2023-01-20

**Authors:** José Pinto, Rafael S Costa, Leonardo Alexandre, João Ramos, Rui Oliveira

**Affiliations:** LAQV-REQUIMTE, Department of Chemistry, NOVA School of Science and Technology, Universidade NOVA de Lisboa, Caparica 2829-516, Portugal; LAQV-REQUIMTE, Department of Chemistry, NOVA School of Science and Technology, Universidade NOVA de Lisboa, Caparica 2829-516, Portugal; LAQV-REQUIMTE, Department of Chemistry, NOVA School of Science and Technology, Universidade NOVA de Lisboa, Caparica 2829-516, Portugal; INESC-ID, Lisboa, Portugal; LAQV-REQUIMTE, Department of Chemistry, NOVA School of Science and Technology, Universidade NOVA de Lisboa, Caparica 2829-516, Portugal; LAQV-REQUIMTE, Department of Chemistry, NOVA School of Science and Technology, Universidade NOVA de Lisboa, Caparica 2829-516, Portugal

## Abstract

**Summary:**

Here, we present *sbml2hyb*, an easy-to-use standalone Python tool that facilitates the conversion of existing mechanistic models of biological systems in Systems Biology Markup Language (SBML) into hybrid semiparametric models that combine mechanistic functions with machine learning (ML). The so-formed hybrid models can be trained and stored back in databases in SBML format. The tool supports a user-friendly export interface with an internal format validator. Two case studies illustrate the use of the *sbml2hyb* tool. Additionally, we describe HMOD, a new model format designed to support and facilitate hybrid models building. It aggregates the mechanistic model information with the ML information and follows as close as possible the SBML rules. We expect the *sbml2hyb* tool and HMOD to greatly facilitate the widespread usage of hybrid modeling techniques for biological systems analysis.

**Availability and implementation:**

The Python interface, source code and the example models used for the case studies are accessible at: https://github.com/r-costa/sbml2hyb.

**Supplementary information:**

Supplementary data are available at *Bioinformatics* online.

## 1 Introduction

Hybrid semiparametric models combine parametric functions (stemming from knowledge) with non-parametric functions (stemming from data) in the same mathematical structure (Thompson and Kramer, 1994). The incorporated parametric functions have a fixed mathematical structure with a fixed number of parameters established from prior knowledge. On the contrary, the non-parametric functions have loose structure without physical meaning. They are frequently needed because critical parts of the model lack fundamental mechanistic knowledge. The number and value of such parameters are not known a priori. They must be established from data during the training process. A typical example is the combination of material balance equations over biochemical species in the form of a system of ordinary differential equations (ODEs)—parametric function easily established from prior knowledge—with an artificial neural network (ANN) to model partially or completely the biologic kinetics—non-parametric function of a more complex part of the model lacking mechanistic understanding (Pinto *et al.*, 2019, 2022).

Hybrid models are currently well established in process systems engineering (von Stosch *et al.*, 2014). The penetration of the hybrid modeling technique in systems biology is however lagging behind. We have previously published hybrid metabolic flux analysis techniques that combine metabolic networks with principal component analysis (Carinhas *et al.*, 2011; Isidro *et al.*, 2016) and partial least squares (Ferreira *et al.*, 2011; Teixeira *et al.*, 2011). The combination of systems of ODEs with ANNs for modeling biochemical networks with intrinsic time delays has been proposed by von Stosch *et al.* (2011). Recent studies have highlighted the need to further integrate systems biology models, particularly genome-scale models (GEMs), with emerging machine learning (ML) techniques (Antonakoudis *et al.*, 2020; Kim *et al.*, 2021; Lewis and Kemp, 2021; Ramos *et al.*, 2022; Vijayakumar *et al.*, 2020; Yang *et al.*, 2019).

A large number of systems biology models, including GEMs, have been developed and stored in databases [e.g. BioModels (Le Novere *et al.*, 2006) and JWS online (Olivier and Snoep, 2004)] in the Systems Biology Markup Language (SBML) format (Hucka *et al.*, 2003). However, existing hybrid modeling tools do not comply with the SBML format. This significantly hinders the interlink between both modeling approaches. Here, we develop the *sbml2hyb* export interface, which allows to convert existing systems biology models into a hybrid model and *vice versa*. Moreover, this work presents a new internal hybrid model format (HMOD) that can be translated to SBML.

**Fig. 1.**
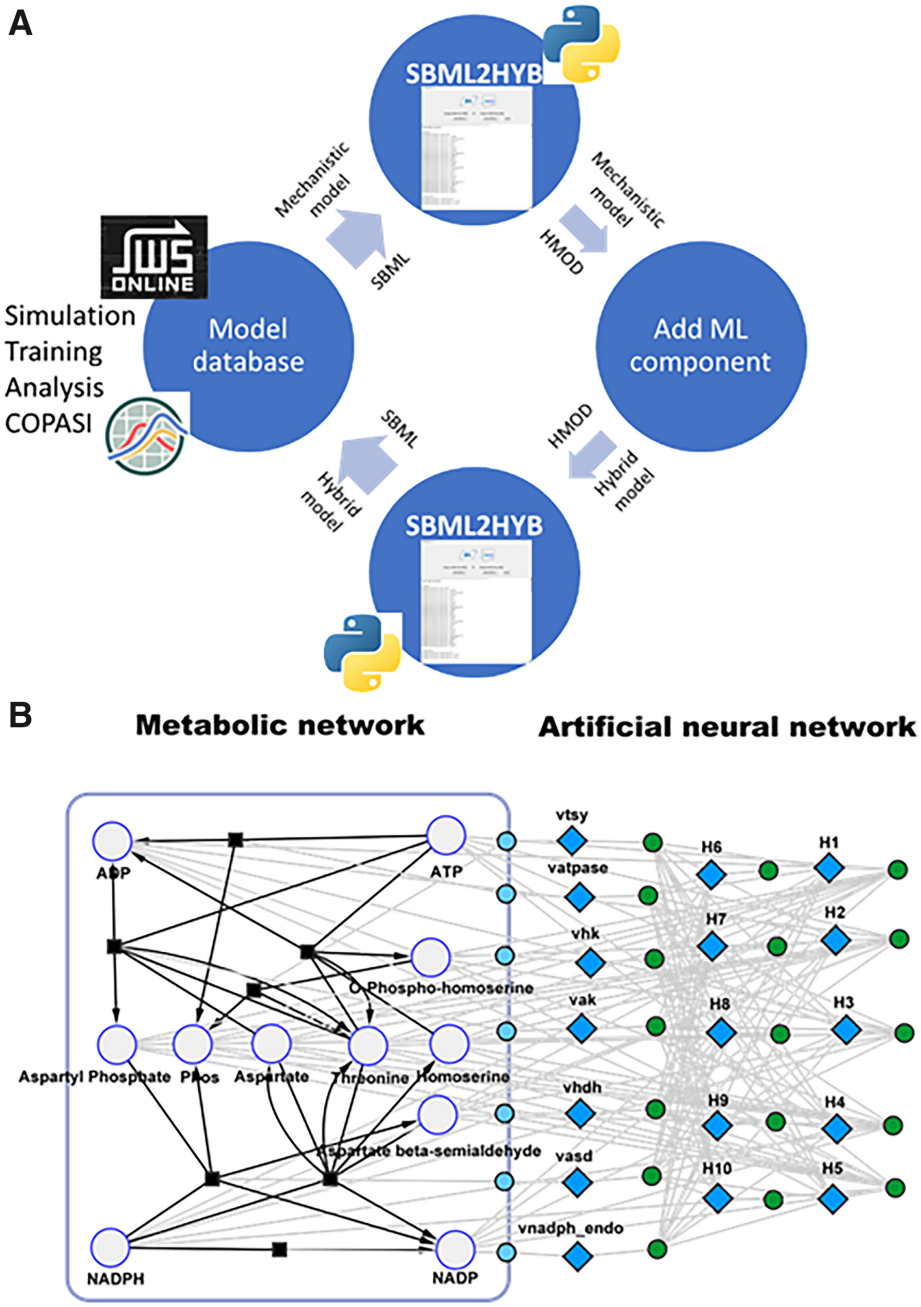
SBML compatible hybrid modeling pipeline. (**A**) Overview of the *sbml2hyb* pipeline. Stored SBML (mechanistic) models in databases are converted to the HMOD format by the *sbml2hyb* tool. The user inputs the information of the ML component in the *sbml2hyb* interface (input/output variables and Keras neural network file in H5 format), which is then automatically added to the HMOD file. The resulting hybrid model HMOD file is reconverted to SBML. The hybrid model in SBML format is stored back in databases; (**B**) Simplified illustration of a hybrid model in SBML format generated by Cytoscape with the cy3sbml app (Konig *et al.*, 2012). On the mechanistic side of the model (left of the image), the larger circles represent the different species of the model, the black squares represent the reactions and the large rectangle refers to the single compartment in this case. On the machine learning side (right of the image), each of the small green circles is a calculation carried out by the ANN, while each blue diamond represents the *results* of those calculations (hidden and output layers). The small blue circles are the final output of the network, which, in this case, is the value that is assigned to each of the reaction rates

Figure 1 shows the pipeline for SBML-compatible hybrid modeling. The proposed workflow enables to convert existing systems biology models stored in databases in SBML format into hybrid models that combine mechanistic equations and ML techniques. SBML is not a common format to encode ML/hybrid models (i.e. multiple formalism models), thus we created an intermediate HMOD format (described below). The user inputs the information of the ML module into the HMOD format (currently limited to feedforward ANNs). The resulting hybrid model in HMOD format is reconverted in SBML and stored back in model databases. Hybrid models in SBML format can be simulated, analyzed, trained with existing tools such as MATLAB and COPASI (Hoops *et al.*, 2006) or special-purpose tools with training algorithms for hybrid systems which are able to read SBML files. To facilitate the conversion between the SBML and HMOD files, a Python-based interface is provided.

## 2 Methods and implementation

### 2.1 Overview of HMOD format

The HMOD format is a text-based file (ASCII) with the list of properties (species, parameters, rates and rules) defining the model that make it easy to parse. This format presents the model components in a similar manner to SBML, by considering any number of species with a certain initial concentration distributed among any number of compartments. These species are then subjected to a list of reactions and rate rules which in turn can be dependent on various parameters and assignment rules.

### 2.2 *SBML2HYB* tool

The *sbml2hyb* tool is implemented in Python 3, along with some open-source libraries for the translations from one file format to another. To parse the SBML files, libSBML (Bornstein *et al.*, 2008) and Python's built-in xml.etree submodule were used. The libSBML translates the MathML expressions to common readable math and *vice versa*. The xml.etree, is a powerful xml parser and builder, which was helpful to get the necessary information and the wanted nodes from the SBML in order to build the HMOD files. These two libraries were used to parse the SBML and get the information to build the HMOD files and also for the MathML translation and SBML file building.

To parse and validate the HMOD files, it was necessary to build a new syntax validator that ensured that the file followed all the rules defined by this file type. The libSBML was also used to translate simple math equations to the MathML used in the SBML files. Hence, two Python modules were constructed (i) a module that receives an SBML, validates the file, and builds the corresponding HMOD file and validates it and (ii) a module that receives an HMOD file and transforms it into an SBML, which is also validated. Any error occurring in the validation phase is displayed in the GUI. This can be because of some xml structure fault, or the HMOD file not respecting the rules defined by the HMOD specification. The GUI was built using the TKinter library (Shipman, 2010). Moreover, the executable Windows file was generated using cx_Freeze (https://cx-freeze.readthedocs.io/en/latest/). The *sbml2hyb* package can be easily extended with additional functionalities that can interoperate with those already implemented.

## 3 Case studies

In order to demonstrate the applicability of the *sbml2hyb* pipeline (Fig. 1), we used two case models taken from the literature. For the first case, the threonine synthesis pathway model of *Escherichia coli* (Chassagnole *et al.*, 2001) in SBML format is freely available at the BioModels Database (BIOMD0000000066) while for the second case [the well-known Park and Ramirez model (1990)] the SBML file was created.

Firstly, the SBML files were converted into HMOD files via the *sbml2hyb* tool. These files contained the necessary information from the SBML in an easy to work format (species, compartments, rules and reactions). Afterwards, the ML component information was added. With the HMOD format containing information from the SBML and ML, it was possible for the parameters and assignments related to ML to be written back into the HMOD file.

Lastly, the obtained HMOD file was translated back through the *sbml2hyb* tool to obtain SBML file with the implemented hybrid model (can be also trained). These SBML files were then uploaded back into the BioModels database (MODEL2207280001 and MODEL2211110001), with the results being very similar to the original mechanistic models (see Supplementary Figs S1 and S2), showing that the hybrid models were successfully trained and turned into an SBML format. All models are available from https://github.com/r-costa/sbml2hyb/tree/main/models.

## 4 Conclusion

Previously published hybrid modeling studies are limited to relatively simple mechanistic models in the hybrid mechanistic/ML ensemble and do not comply with the SBML format. In this paper, we presented *sbml2hyb*, a Python application for SBML-compatible hybrid model encoding. It is easy to use and allows creating further extensions that can easily incorporate new model components. We expect the *sbml2hyb* tool to greatly facilitate the extension of existing SBML mechanistic models to the hybrid mechanistic/ML approach. All in all, the possibility to encode hybrid models in SBML format will accelerate the adoption of the hybrid modeling techniques by the systems biology community.

## Supplementary Material

btad044_Supplementary_Data
