# Supplementary material for: SBML2HYB: a Python interface for SBML compatible hybrid modeling
Source: Bioinformatics. 2023 Jan 20;39(1):btad044. doi: 10.1093/bioinformatics/btad044 (PMC9889961; doi:10.1093/bioinformatics/btad044)
Supplement: btad044_Supplementary_Data [file btad044_supplementary_data.docx]

Supplementary Material for *SBML2HYB: a Python interface for SBML compatible hybrid modelling*

Supplementary Figures


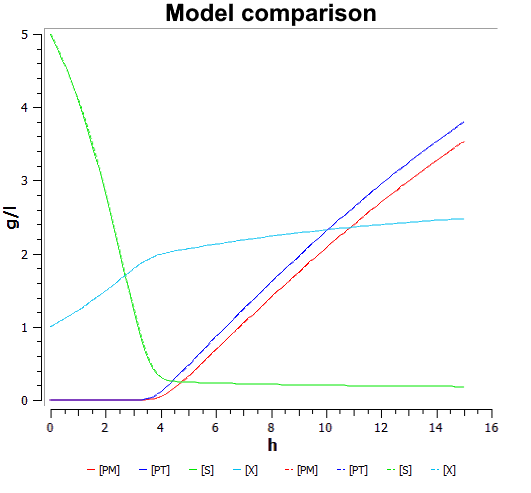


**Figure S1:** Comparison of simulated metabolite concentrations over time for the mechanistic and the hybrid SBML *Park&Ramirez* model (after training). The filled line corresponds to the mechanistic model while the dashed line corresponds to the hybrid model (lines overlapped). Parameter identification was carried out in MATLAB using the Levenberg-Marquardt algorithm to fit a set of training data and then tested in a different set for validation. Simulations obtained from the COPASI software.


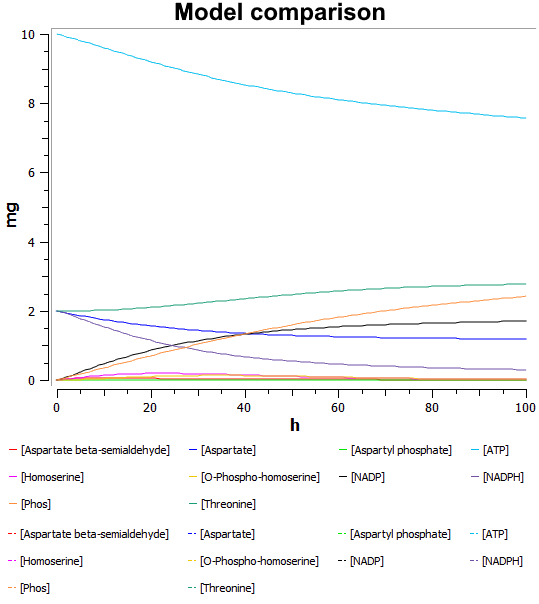


**Figure S2:** Comparison of simulated metabolite weights over time for the mechanistic and the hybrid SBML *Chassagnole* model (after training). The filled line corresponds to the mechanistic model while the dashed line corresponds to the hybrid model (lines overlapped). Parameter identification was carried out in MATLAB using the Levenberg-Marquardt algorithm to fit a set of training data and then tested in a different set for validation. Simulations obtained from the COPASI software.
